# Impact of Inter-Set Short Rest Interval Length on Inhibitory Control Improvements Following Low-Intensity Resistance Exercise in Healthy Young Males

**DOI:** 10.3389/fphys.2021.741966

**Published:** 2021-11-22

**Authors:** Keigo Tomoo, Tadashi Suga, Kento Dora, Takeshi Sugimoto, Ernest Mok, Hayato Tsukamoto, Shingo Takada, Takeshi Hashimoto, Tadao Isaka

**Affiliations:** ^1^Faculty of Sport and Health Science, Ritsumeikan University, Kusatsu, Japan; ^2^Research Organization of Science and Technology, Ritsumeikan University, Kusatsu, Japan; ^3^Department of Sports Education, Faculty of Lifelong Sport, Hokusho University, Ebetsu, Japan

**Keywords:** cognitive function, brain health, lactate, electromyography, perceived exertion, exercise adherence

## Abstract

The length of rest interval between sets (i.e., inter-set rest interval) is an important variable for resistance exercise program. However, the impact of the inter-set rest interval on improvements in cognitive function following resistance exercise remains unknown. In this study, we compared the effect of short rest interval (SRI) vs. long rest interval (LRI) protocols on post-exercise cognitive inhibitory control (IC) improvements induced by low-intensity resistance exercise. Twenty healthy, young males completed both SRI and LRI sessions in a crossover design. The bilateral knee extensor low-intensity resistance exercise was programed for six sets with 10 repetitions per set using 40% of one-repetition maximum. The inter-set rest interval lengths for SRI and LRI protocols were set for 1 and 3min, respectively. The color-word Stroop task (CWST) was administrated at six time points: baseline, pre-exercise, immediate post-exercise, and every 10min during the 30-min post-exercise recovery period. The levels of blood lactate, which may be an important determinant for improving IC, throughout the 30-min post-exercise recovery period were significantly higher following SRI protocol than following LRI protocol (*p*=0.002 for interaction effect). In line with this result, large-sized decreases in the reverse-Stroop interference score, which represent improved IC, were observed immediately after SRI protocol (*d*=0.94 and 0.82, respectively, vs. baseline and pre-exercise) as opposed to the moderate-sized decreases immediately after LRI protocol (*d*=0.62 and 0.66, respectively, vs. baseline and pre-exercise). Moreover, significant decreases in the reverse-Stroop interference score were observed from 10 to 30min after SRI protocol (all *p*s<0.05 vs. baseline and/or pre-exercise), whereas no such decrease was observed after LRI protocol. Furthermore, the degree of decreases in the reverse-Stroop interference score throughout the 30-min post-exercise recovery period was significantly greater in SRI protocol than in LRI protocol (*p*=0.046 for interaction effect). We suggest that the SRI protocol is more useful in improving post-exercise IC, potentially *via* greater circulating lactate levels, compared to the LRI protocol. Therefore, the inter-set rest interval length may be an important variable for determining the degree of cognitive function improvements following resistance exercise in healthy young males.

## Introduction

Skeletal muscle weakness, as seen in decreased muscle mass and strength, is a prominent factor that indicates poor prognosis in older individuals and patients with chronic diseases (Ruiz et al., 2008). Many people with skeletal muscle weakness also present decreased cognitive function (Chang et al., 2016), which is also a poor prognostic factor (DeCarli, 2003). Because a complication of skeletal muscle weakness and decreased cognitive function additively exacerbates physical inactivity (Atkinson et al., 2005), resolution of this public health problem worldwide is now urgent.

Resistance exercise is the most beneficial strategy for increasing skeletal muscle size and strength (Williams et al., 2007; American College of Sports Medicine, 2009). Additionally, long-term intervention of resistance exercise improves cognitive function in healthy young and older individuals (Northey et al., 2018; Ludyga et al., 2020). Furthermore, long-term resistance exercise is effective in improving cognitive function in patients with chronic diseases, including cognitive impairment (e.g., mild cognitive impairment; Northey et al., 2018). Therefore, resistance exercise has recently been recognized as an effective strategy for enhancing skeletal muscle and cognitive health in various populations.

The inhibitory control (IC) is a specific cognitive function that is defined as the suppression of behavior in response to either internal or external stimuli (Ozonoff and Strayer, 1997). This is necessary to prevent the implementation of an unrequired action during cognitive processing (Coxon et al., 2007). Therefore, the IC is important for all cognitive processes (Smith and Jonides, 1999).

Some meta-analytical investigations have suggested that improvement in cognitive function induced by an acute bout of exercise was greater in moderate-intensity exercise than in low- and high-intensity exercises (Chang et al., 2012; McMorris and Hale, 2012). Thus, the relationship between exercise intensity and cognitive function improvement generally appears to have an inverted-U shape (Tomporowski, 2003). However, improvements in post-exercise cognitive function, including IC, when aerobic exercise is performed may be affected not only by exercise intensity but also by other exercise variables such as exercise duration and exercise mode (e.g., continuous exercise vs. interval exercise; Chang et al., 2012; Moreau and Chou, 2019; Oberste et al., 2019; Ai et al., 2021). Furthermore, post-exercise cognitive function improvements may be delayed following high-intensity exercise (Chang et al., 2012; Moreau and Chou, 2019; Ai et al., 2021). More importantly, most previous studies that were included in the meta-analytical investigations examined using aerobic exercise (Chang et al., 2012; McMorris and Hale, 2012; Moreau and Chou, 2019; Oberste et al., 2019; Ai et al., 2021). Altogether, the impacts of exercise variables, such as exercise intensity, on improvements in cognitive function induced by an acute bout of resistance exercise are poorly understood.

We and others have previously reported that some resistance exercise protocols, particularly those with high-intensity loads, effectively improve post-exercise IC in various populations (e.g., Brush et al., 2016; Johnson et al., 2016; Tsukamoto et al., 2017a; Tomoo et al., 2020). Furthermore, our previous studies found that this resistance exercise-induced positive effect on IC can also be observed using low-intensity loads (Tsukamoto et al., 2017a; Tomoo et al., 2020). Therefore, the relationship between exercise intensity and IC improvements induced by resistance exercise may be slightly different from that induced by aerobic exercise.

The low-intensity resistance exercise is known to be a higher applicability, because of lower loads to the body on cardiovascular and musculoskeletal system, than high-intensity resistance exercise in some populations, especially older individuals and patients with chronic disease (Williams et al., 2007). However, our previous study reported that the degree of IC improvement immediately after resistance exercise was greater for high-intensity resistance exercise than for low-intensity resistance exercise (Tsukamoto et al., 2017a). This suggests that a conventional protocol with lower-intensity loads may be inadequate for improving post-exercise IC. Therefore, it would be useful to identify effective strategies for enhancing IC improvement following low-intensity resistance exercise.

The length of rest interval between sets (i.e., inter-set rest interval) is an important variable for resistance exercise program. Previous studies have reported that short rest interval (SRI) protocol may be more useful to enhancing resistance training-induced muscle adaptation (e.g., muscle hypertrophy and strength gain) than long rest interval (LRI) protocol (Takarada and Ishii, 2002; Goto et al., 2004). This positive effect of long-term resistance exercise with SRI may be at least partially due to increased levels of by-products such as lactate during an acute bout of this protocol (Kraemer et al., 1990, 1993). We have previously demonstrated that the increased levels of circulating lactate induced by aerobic exercise are related to the degree of post-exercise IC improvements (Tsukamoto et al., 2016b; Hashimoto et al., 2018), potentially by increasing cerebral lactate metabolism (Hashimoto et al., 2018). Based on these findings, we hypothesized that the SRI protocol would be more effective in enhancing low-intensity resistance exercise-induced IC improvements, potentially with greater circulating lactate levels, compared to the LRI protocol. To test this hypothesis, we compared the effect of SRI vs. LRI protocols on IC improvements following low-intensity resistance exercise.

## Materials and Methods

### Participants

Twenty healthy, young males (age: 20.7±0.5years, body height: 172.8±1.0cm, body weight: 62.9±1.2kg) participated in this study. The subjects were recruited *via* flyers or referral from participants who were previously included in the study. The subjects were recreationally active and participated in physical exercise (e.g., resistance exercise and/or aerobic exercise) for 2–4h per week. The subjects were free of any known neurological, cardiovascular, and pulmonary disorders, as well as free from color-blindness and abnormal vision. All subjects provided written informed consent upon having the experimental procedures and potential risks described to them. The study was approved by the Ethics Committee of Ritsumeikan University and conducted according to the Declaration of Helsinki.

### Experimental Design

**Figure 1:**
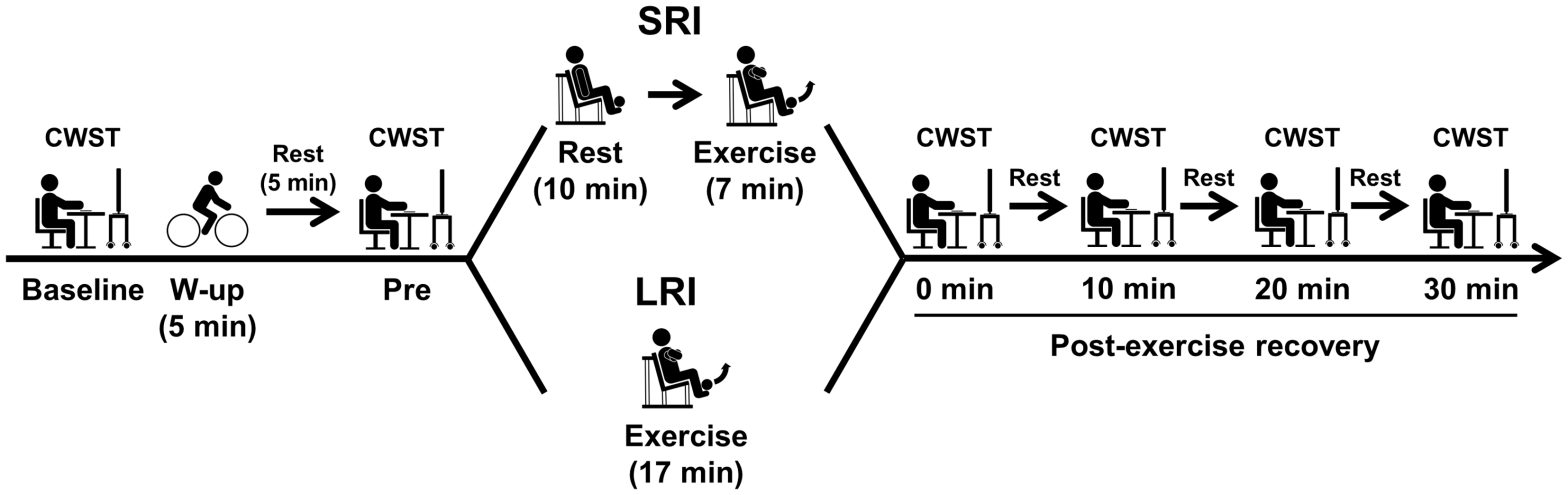
Experimental procedures for experimental sessions of inter-set short rest interval (SRI) and long rest interval (LRI) protocols. The bilateral knee extensor resistance exercise for both SRI and LRI protocols was programmed for six sets with 10 repetitions per set. The inter-set rest interval lengths for SRI and LRI were set for 1 and 3min, respectively. The exercise load for both protocols was applied for 40% of one-repetition maximum. During experimental session, the color-word Stroop task (CWST) was administered at six time points: baseline, pre-exercise (i.e., Pre), immediately after exercise (i.e., 0min at post-exercise recovery), and every 10min during the 30-min post-exercise recovery period.

Experimental procedures of this study are presented in Figure 1. On the day of familiarization visit, subjects practiced the three types of the color-word Stroop task (CWST) for each a minimum of 10 times until they achieved consistent scores, as in our previous studies (Tsukamoto et al., 2016a,b, 2017a,b, 2018; Hashimoto et al., 2018; Tanaka et al., 2018; Sugimoto et al., 2020, 2021; Tomoo et al., 2020). During the familiarization day, the subjects also completed measurement of one-repetition maximum (1-RM) of the bilateral knee extension, which was used to calculate low-intensity exercise load for both SRI and LRI protocols.

On the two experiment days, the subjects visited after fasting overnight (i.e., abstinence from food for 12h) and avoiding strenuous physical activity for 24h prior to the experiment. The subjects also abstained from caffeine and alcohol for 12h prior to the experiment and were not taking any medications that would affect their cognitive performances.

In the experiment session, the subjects first practiced the three CWST types for each a minimum of five times before experimental session to minimize the learning effect, as in our previous studies (Tsukamoto et al., 2016a,b, 2017a,b, 2018; Hashimoto et al., 2018; Tanaka et al., 2018; Sugimoto et al., 2020, 2021; Tomoo et al., 2020). After the CWST practice, the subjects rested for 5min and then performed the baseline CWST.

Next, the subjects performed a warm-up exercise at 50W for 5min using a bicycle ergometer (Life Fitness; Schiller Park, IL, United States), as in our previous studies (Tsukamoto et al., 2017a; Tomoo et al., 2020). Our previous studies have confirmed that duration and intensity of the warm-up exercise do not result in positive effect on IC (Tsukamoto et al., 2017a; Tomoo et al., 2020). After the warm-up exercise, the subjects rested for 5min and then performed the pre-exercise CWST to confirm that the warm-up exercise did not affect IC.

Subsequently, the subjects completed either SRI or LRI session. The CWST was performed again immediately after the completion of the exercise session and then repeated three times at 10-min intervals during the 30-min post-exercise recovery period to evaluate the sustainable effects of post-exercise IC improvements, similar to our previous studies (Tsukamoto et al., 2016a,b, 2017b, 2018; Hashimoto et al., 2018; Tanaka et al., 2018; Sugimoto et al., 2020; Tomoo et al., 2020).

Heart rate (HR), ratings of perceived exertion (RPE) and leg discomfort, and electromyography (EMG) activities of the quadriceps femoris muscles during exercise were measured in every set to determine the levels of cardiovascular, perceptual, and neuromuscular responses. Fingertip blood samples were collected immediately before all six CWSTs to assess the effects of metabolic conditions on IC. The Felt Arousal Scale (FAS) and Visual Analogue Scale (VAS) were measured immediately after all CWSTs to assess the effects of psychological conditions on IC.

In a preliminary study, to account for the learning effect on IC improvements following exercise, we examined the effect of sitting at rest for 17min on IC in eight healthy young males. According to the experimental design of the main study, the CWST was administrated at six time points: baseline, before sitting rest, immediately after sitting rest, and every 10min during 30-min after sitting rest. This result showed that response times and accuracies of the three CWST types and the reverse-Stroop interference score (i.e., IC) did not significantly differ among all six time points (data not shown). Therefore, we considered that the results of low-intensity resistance exercise-induced IC improvements obtained in this study were not attributable to a learning effect.

### Experimental Protocols

The bilateral knee extensor low-intensity resistance exercise for both SRI and LRI sessions was programmed for six sets with 10 repetitions per set using a leg extension machine (Life Fitness; Schiller Park, IL, United States). The lengths of inter-set rest intervals for SRI and LRI protocols were set for 1 and 3min, respectively, according to previous studies (Kraemer et al., 1990, 1993). An exercise load for both protocols was applied to 40% of 1-RM, as in our previous study (Tsukamoto et al., 2017a). The two experimental sessions were performed at approximately the same time (±1h) in the morning, as separated by 1week.

### One-Repetition Maximum

The 1-RM was measured on the familiarization visit and was determined by a successful concentric contraction of the bilateral knee extension to calculate an exercise load for both SRI and LRI protocols, as previously described (Suga et al., 2009; Tsukamoto et al., 2017a; Tomoo et al., 2020). The mean 1-RM of the bilateral knee extension in all subjects was 117±5kg. The mean 40% 1-RM load for both SRI and LRI in all subjects was 46±2kg.

### Heart Rate

HR was measured continuously *via* telemetry (RS400; Polar Electro Japan, Tokyo, Japan). Peak HR was collected every set during exercise session, and the mean value of all six sets was calculated for analysis.

### Perceived Exertion

The Borg’s 15-point scale-measured RPE was collected to assess perceived exertion during exercise, which ranges from 6 (no exertion) to 20 (maximal exertion; Borg, 1982). The Borg’s category-ratio scale was collected to assess the leg discomfort during exercise, which ranges from 0 (nothing at all) to 10 (very, very strong; Borg, 1982). Both scales were collected every set during exercise session, and the mean values of all six sets for each were calculated for analysis.

### Quadriceps Femoris Electromyography

Prior to the application of electrodes, the subject’s skin was shaved, abraded, and cleaned with alcohol to minimize skin impedance. EMG signals were recorded with surface electrodes from the quadriceps vastus lateralis, vastus medialis, and rectus femoris muscles. The EMG signals were amplified 1,000 times, band-pass filtered between 10 and 500Hz, and sampled at 1,000Hz (MQ-Air; Kissei Comtech, Nagano, Japan). The EMG activities of the three quadriceps femoris muscle during exercise were quantified as the integration of the rectified EMG (iEMG) over 1s. Thereafter, the iEMG were normalized to the highest iEMG (the average value over 1s) that was obtained during the two trials of knee extension maximal voluntary contractions, which was measured after the CWST was completed at the 30-min post-exercise recovery period. Peak iEMGs of each muscle were calculated from the last five repetitions during the first and final sets. Mean iEMG values of the five repetitions during the first and final sets for each muscle were calculated for analyses of this study.

### Blood Metabolites

Blood glucose and lactate levels were measured using a glucose (Medisafe FIT Blood Glucose Meter; Terumo, Tokyo, Japan) and lactate analyzer (Lactate Pro 2; Arkray, Kyoto, Japan), respectively.

### Color-Word Stroop Task

The detailed methods of the CWST have been described in our previous studies (Tsukamoto et al., 2016a,b, 2017a,b, 2018; Hashimoto et al., 2018; Tanaka et al., 2018; Sugimoto et al., 2020, 2021; Tomoo et al., 2020). The stimuli words were four color names (i.e., RED, YELLOW, GREEN, and BLUE), and they were presented on a 98-inch display. The three types of the CWST consisted of two color text tasks (i.e., congruent and incongruent tasks) and one control black text task (i.e., neutral task). The congruent task, which is a facilitated task, displayed the color names presented in the same-colored text. The neutral task, which is a control task, displayed the color names presented in black text. The incongruent task, which is an interference task, displayed the color names presented in a different-colored text. The subjects were randomly provided the three CWST types, and it was repeated three times (i.e., the three trials per each CWST type). The intervals between each trial were set at 1s. During the CWST, the subjects did not receive feedback regarding their CWST performances (e.g., reaction time and response accuracy) unless there were clear technical errors. All CWSTs throughout the experimental session were measured by the same examiner who was familiar with the procedure.

All CWST trials consisted of 24 stimulus words. The reaction time was evaluated as the total time taken to complete the 24 stimulus words per trial. The response accuracy was evaluated as the error rate of the 24 stimulus words per trial. The mean values of the reaction times and response accuracies on three trials of each CWST type were used for analyses of this study. The IC was assessed using the reverse-Stroop interference score, which was calculated as the difference between the reaction times of the neutral and incongruent tasks. The reverse-Stroop interference score was calculated as [(reaction time of incongruent task – reaction time of neutral task)/reaction time of neutral task×100] (Ikeda et al., 2010). The mean value of the reverse-Stroop interference score on the three trials was used for analyses of this study.

### Psychological Conditions for the CWST

FAS is a six-point, single-item scale ranging from 1 (low-arousal) to 6 (high arousal; Svebak and Murgatroyd, 1985). VAS consisted of questions of three psychological types that assess mental fatigue, motivation, and concentration. Each VAS was labeled from 0mm (i.e., not at all) to 100mm (i.e., extremely). Subjects drew lines to indicate their response.

### Statistical Analysis

All data are expressed as mean±standard error of the mean. Comparisons of mean values of HR and perceived exertion parameters (i.e., RPE and leg discomfort) during SRI and LRI protocols were performed using a paired Student’s *t* test. Changes in EMG activities of the three quadriceps femoris muscles during the two protocols were analyzed using two-way (two conditions×two times) repeated-measures ANOVA. Changes in the CWST-related parameters (i.e., reaction time, response accuracy, and the reverse-Stroop interference score) throughout the two experimental sessions were analyzed using two-way (two conditions×six times) ANOVA. If the sphericity assumption was not met, Greenhouse–Geisser corrections were used. Specific differences were identified with a paired Student’s *t* test or a Bonferroni *post hoc* test. The statistical significance level was defined at *p*<0.05. All statistical analyses were conducted using IBM SPSS software (Ver. 19.0, IBM Corp, NY, United States).

The Cohen’s *d* effect size using the pooled SD was calculated as effect size to determine the magnitude of the difference in measured variables between conditions or time points. This effect size was interpreted as small (0.20–0.49), medium (0.50–0.79) and large (>0.80; Cohen, 1992). Partial eta squared (*η*_p_^2^) was calculated as effect size to determine the magnitude of main effects of condition and time or interaction effect.

To determine the statistical power and sensitivity of repeated measures effects obtained in this study, we performed repeated measures within-factor *post hoc* power and sensitivity analyses using an effect size (*ƒ*) of 0.65 that was calculated from *η*_p_^2^ (i.e., 0.30) of the time main effect of one-way repeated-measures ANOVA (i.e., six measurements) for a main outcome measure (i.e., the reverse-Stroop interference score for SRI), as computed using G*Power 3.1.9.2 (Faul et al., 2007). The *post hoc* power analysis, performed with an actual alpha of <0.001, correlation between repeated measures of 0.56, and nonsphericity correction of 0.68, revealed adequate statistical power (1-β) of >0.99. The *post hoc* sensitivity analysis, performed with this power and other variables same as the *post hoc* power analysis, revealed sufficient statistical sensitivity to detect a large effect size (*ƒ*) of 0.48 (Cohen, 1992). The necessary number of subjects to obtain significant repeated measures effects was 5, based on an alpha of 0.50 and power (1-β) of 0.80. Therefore, this study was performed with a sufficient sample size.

## Results

### Measured Variables During Exercise

Mean HR during exercise did not differ significantly between SRI and LRI protocols (107±2 and 105±2bpm, respectively; *p*=0.097, *d*=0.26). In contrast, mean values of RPE and leg discomfort during low-intensity resistance exercise were significantly higher with SRI protocol than with LRI protocol (RPE: 12.7±0.3 vs. 12.1±0.3, respectively; leg discomfort: 4.4±0.3 vs. 3.8±0.3, respectively; both *p*s<0.01, *d*=0.49 and 0.45, respectively).

**Figure 2:**
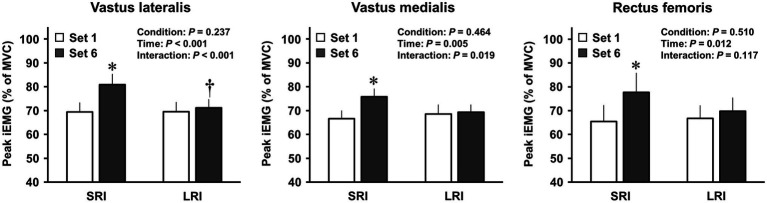
Changes in activation levels of the quadriceps femoris muscles during SRI and RI protocols. Neural activation levels of the quadriceps femoris muscles during the knee extensor resistance exercise were evaluated using the integrated electromyography (iEMG). The peak iEMGs of the three quadriceps femoris muscles during the first and final sets of SRI and LRI protocols were calculated from the last five repetitions, and the five repetitions for each muscle were calculated for the analysis. Values are presented as mean±standard error of the mean. ^*^Significant difference (*p*<0.001) between the first and final sets during SRI protocol. ^†^Significant difference (*p*<0.05) between SRI and LRI protocols.

Changes in EMG activities of the three quadriceps femoris muscles during the first and last sets in SRI and LRI protocols are shown in Figure 2. Analyses of EMG activities for all three muscles revealed significant main effects for time (vastus lateralis: *F*_(1, 19)_=29.70, *p*<0.001, *η*_p_^2^=0.61; vastus medialis: *F*_(1, 19)_=10.24, *p*=0.005, *η*_p_^2^=0.35; rectus femoris: *F*_(1, 19)_=7.82, *p*=0.012, *η*_p_^2^=0.29); however, there were no significant main effects for condition (vastus lateralis: *F*_(1, 19)_=1.49, *p*=0.237, *η*_p_^2^=0.07; vastus medialis: *F*_(1, 19)_=0.56, *p*=0.464, *η*_p_^2^=0.03; rectus femoris: *F*_(1, 19)_=0.45, *p*=0.510, *η*_p_^2^=0.02). *Post hoc* comparisons for these time main effects indicated that the EMG activities for each muscle were significantly higher during the last set than during the first set (all *p*s<0.05). The EMG activities of all three quadriceps femoris muscles significantly increased during the last set in SRI protocol, but not in LRI protocol, compared with those during the first set (all *p*s<0.05, *d*=0.36–0.61). Additionally, analyses of EMG activities for the vastus lateralis and vastus medialis revealed significant interaction effects (vastus lateralis: *F*_(1, 19)_=18.46, *p*<0001, *η*_p_^2^=0.49; vastus medialis: *F*_(1, 19)_=6.61, *p*=0.019, *η*_p_^2^=0.26); however, there was no significant interaction effect for the rectus femoris (*F*_(1, 19)_=2.70, *p*=0.117, *η*_p_^2^=0.12). The vastus lateralis EMG activity during the last set was significantly higher for SRI protocol than for LRI protocol (*p*=0.026, *d*=0.54). A trend against such a significance was observed for the vastus medialis EMG activity (*p*=0.062, *d*=0.44).

### Changes in Blood Metabolites Throughout Experimental Session

**Figure 3:**
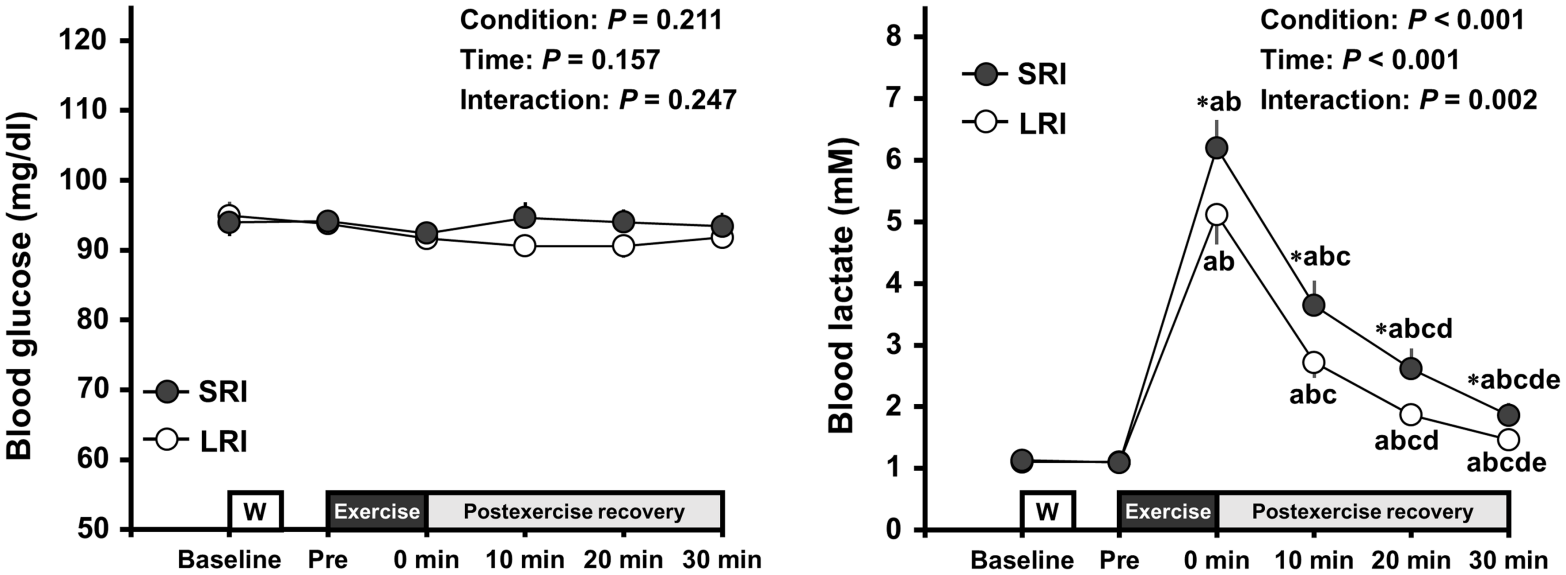
Changes in blood glucose and lactate levels throughout SRI and RI sessions. Values are presented as mean±standard error of the mean. ^*^Significant difference (*p*<0.05) between SRI and LRI sessions. ^a^*p*<0.05 vs. Baseline, ^b^*p*<0.05 vs. Pre, ^c^*p*<0.05 vs. immediately after exercise (i.e., 0min), ^d^*p*<0.05 vs. 10min after exercise, and ^e^*p*<0.05 vs. 20min after exercise.

Changes in blood metabolites throughout SRI and LRI sessions are presented in Figure 3. Blood glucose analysis revealed no significant main effects for condition and time or no significant interaction effect (condition main effect: *F*_(1, 19)_=1.68, *p*=0.211, *η*_p_^2^=0.08; time main effect: *F*_(5, 95)_=1.64, *p*=0.157, *η*_p_^2^=0.08; interaction effect: *F*_(2.51, 47.77)_=1.44, *p*=0.247, *η*_p_^2^=0.07). In contrast, blood lactate analysis revealed significant main effects for condition (*F*_(1, 19)_=18.24, *p*<0.001, *η*_p_^2^=0.49) and time (*F*_(1.18, 22.34)_=94.65, *p*<0.001, *η*_p_^2^=0.83) and a significant interaction effect (*F*_(2.21, 41.90)_=7.19, *p*=0.002, *η*_p_^2^=0.28). Blood lactate significantly increased immediately after both SRI and LRI protocols compared to that before each exercise (all *p*s<0.001, *d*=2.60–3.52 vs. baseline and pre-exercise). The increased lactate remained significant throughout the 30-min post-exercise recovery period for both protocols. The blood lactate levels throughout the 30-min post-exercise recovery period were significantly higher for SRI protocol than for LRI protocol (all *p*s<0.05, *d*=0.47–0.56).

### Changes in the CWST-Measured Inhibitory Control Throughout Experimental Session

Reaction times and response accuracies for three CWST types throughout SRI and LRI sessions are summarized in Table 1. Reaction time analysis for incongruent task revealed a significant main effect for time (*F*_(5, 95)_=11.10, *p*<0.001, *η_p_*^2^=0.37); however, there was no significant main effect for condition (*F*_(1, 19)_=2.90, *p*=0.105, *η_p_*^2^=0.13) and no significant interaction effect (*F*_(5, 95)_=1.86, *p*=0.109, *η_p_*^2^=0.09). *Post hoc* comparison for this time main effect indicated that incongruent reaction time significantly shortened immediately after exercise compared with that before exercise (both *p*s≤0.001 vs. baseline and pre-exercise). The shortened incongruent reaction time remained significant until 20min after exercise (all *p*s<0.05 vs. baseline and/or pre-exercise). Analyses of reaction times for congruent and neutral tasks revealed no significant main effects for condition and time and no significant interaction effects (congruent task: *F*_(1, 19)_=1.67, *p*=0.212, *η_p_*^2^=0.08 for condition main effect, *F*_(5, 95)_=2.08, *p*=0.074, *η_p_*^2^=0.10 for time main effect, *F*_(5, 95)_=1.26, *p*=0.289, *η_p_*^2^=0.06 for interaction effect; neutral task: *F*_(1, 19)_=0.91, *p*=0.352, *η_p_*^2^=0.05 for condition main effect, *F*_(5, 95)_=1.77, *p*=0.126, *η_p_*^2^=0.09 for time main effect, *F*_(5, 95)_=0.56, *p*=0.729, *η_p_*^2^=0.03 for interaction effect). Additionally, response accuracy analyses for all three CWST types revealed no significant main effects for condition and time and no significant interaction effects (congruent task: *F*_(1, 19)_=0.20, *p*=0.657, *η_p_*^2^=0.01 for condition main effect, *F*_(5, 95)_=0.25, *p*=0.940, *η_p_*^2^=0.01 for time main effect, *F*_(5, 95)_=0.48, *p*=0.793, *η_p_*^2^=0.02 for interaction effect; neutral task: *F*_(1, 19)_=1.96, *p*=0.178, *η_p_*^2^=0.09 for condition main effect, *F*_(5, 95)_=0.73, *p*=0.602, *η_p_*^2^=0.04 for time main effect, *F*_(5, 95)_=0.94, *p*=0.460, *η_p_*^2^=0.05 for interaction effect; incongruent task: *F*_(1, 19)_=0.95, *p*=0.342, *η_p_*^2^=0.05 for condition main effect, *F*_(5, 95)_=0.93, *p*=0.463, *η_p_*^2^=0.05 for time main effect, *F*_(5, 95)_=0.83, *p*=0.534, *η_p_*^2^=0.04 for interaction effect).

**Table 1:** Changes in reaction times and response accuracies of the three CWSTs throughout experimental sessions of SRI and LRI protocols.

	Time points						*p*		
	Baseline	Pre-EX	Post-EX 0	Post-EX 10	Post-EX 20	Post-EX 30	Condition	Time	Interaction
**Reaction time (msec)**									
**Congruent task**									
SRI	9,788 ± 292	9,740 ± 359	9,380 ± 291	9,288 ± 324	9,450 ± 375	9,593 ± 336	0.212	0.074	0.289
LRI	9,740 ± 316	9,786 ± 282	9,528 ± 338	9,753 ± 345	9,786 ± 323	9,923 ± 355			
**Neutral task**									
SRI	10,160 ± 368	10,043 ± 341	9,817 ± 314	9,756 ± 314	9,935 ± 381	9,962 ± 358	0.352	0.126	0.729
LRI	10,138 ± 355	10,204 ± 334	9,983 ± 297	10,029 ± 336	10,039 ± 349	10,160 ± 326			
**Incongruent task**									
SRI	11,219 ± 417	11,070 ± 368	10,432 ± 345^a^^,^^b^	10,273 ± 340^a^^,^^b^	10,555 ± 432^a^	10,604 ± 408^a^	0.105	**<0.001**	0.109
LRI	11,182 ± 368	11,255 ± 362	10,784 ± 341	10,794 ± 337	11,022 ± 396	11,212 ± 387			
**Response accuracy (%)**									
**Congruent task**									
SRI	95 ± 1	96 ± 1	96 ± 1	96 ± 1	96 ± 1	96 ± 1	0.657	0.940	0.793
LRI	97 ± 1	96 ± 1	96 ± 1	96 ± 1	96 ± 1	96 ± 1			
**Neutral task**									
SRI	96 ± 1	96 ± 1	95 ± 1	96 ± 1	96 ± 1	96 ± 1	0.178	0.602	0.460
LRI	97 ± 1	97 ± 1	96 ± 1	96 ± 1	96 ± 1	98 ± 0			
**Incongruent task**									
SRI	95 ± 1	96 ± 1	95 ± 1	97 ± 1	96 ± 1	96 ± 1	0.342	0.463	0.534
LRI	96 ± 1	95 ± 1	95 ± 1	96 ± 1	95 ± 1	96 ± 1			

Values are presented as mean±standard error of the mean. Pre-EX; before exercise, Post-EX 0; immediately after exercise, Post-EX 10; 10-min at post-exercise recovery period, Post-EX 20; 20-min at post-exercise recovery period, and Post-EX 30; 30-min at post-exercise recovery period. Bold value of *p* indicates a significant main time effect for incongruent reaction time.

a *p*<0.05 vs. Baseline.

b *p*<0.05 vs. Pre-EX.

**Figure 4:**
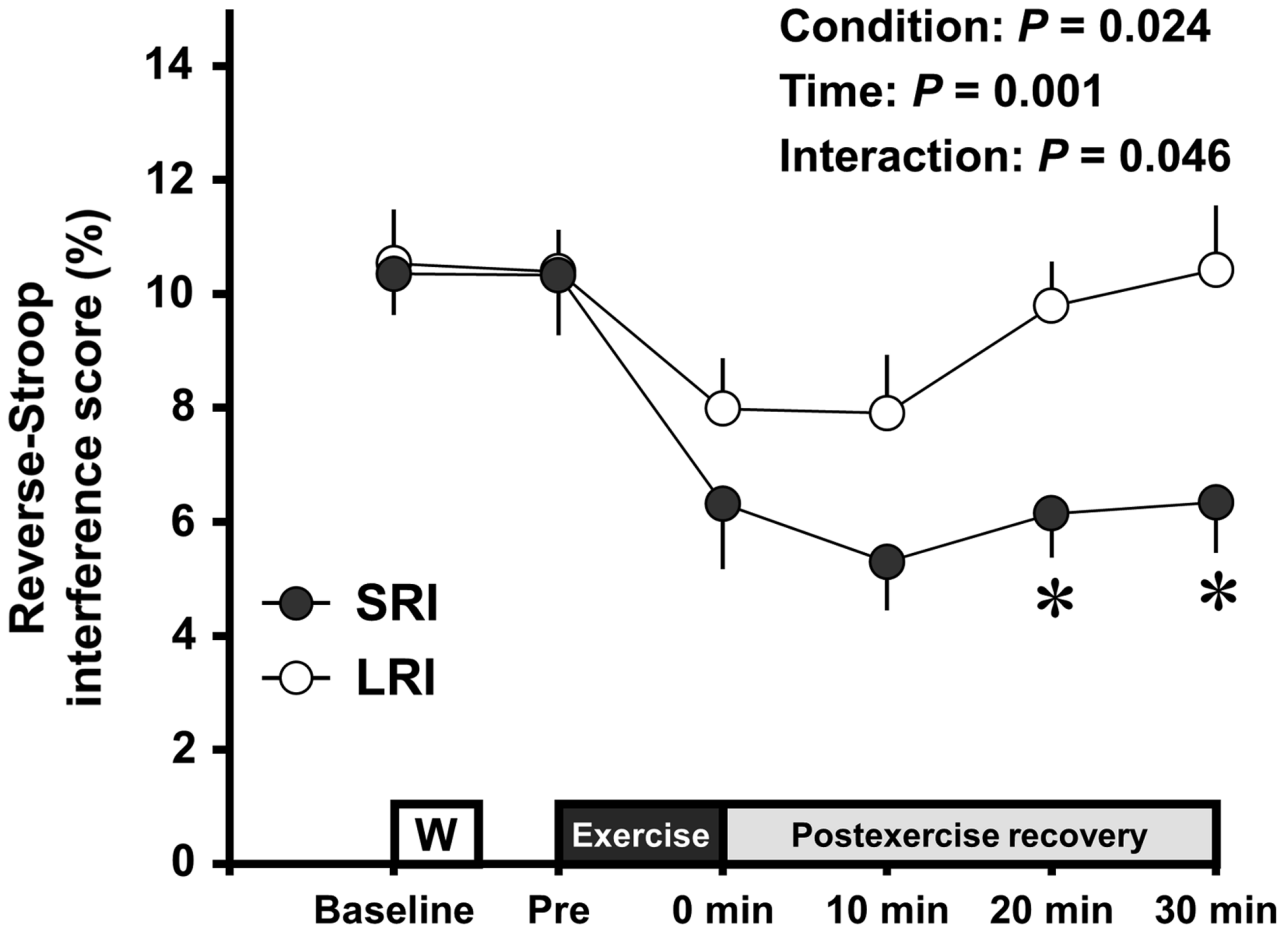
Change in the reverse-Stroop interference scores throughout SRI and LRI sessions. Values are presented as mean±standard error of the mean. ^*^Significant difference (*p*<0.05) between SRI and LRI sessions. Individual *p* values and effect sizes [95% CI] among time points are summarized in Table 2.

Changes in the reverse-Stroop interference scores throughout SRI and LRI sessions are presented in Figure 4. The reverse-Stroop interference score analysis revealed significant main effects for condition (*F*_(1, 19)_=5.98, *p*=0.024, *η*_p_^2^=0.24) and time (*F*_(2.78, 52.79)_=6.30, *p*=0.001, *η*_p_^2^=0.25) and a significant interaction effect (*F*_(5, 95)_=2.58, *p*=0.031, *η*_p_^2^=0.12). The reverse-Stroop interference score significantly decreased after SRI protocol but not after LRI protocol, compared with that before exercise (i.e., see Table 2). Effect sizes for comparison of the reverse-Stroop interference scores before (i.e., baseline and pre-exercise) and after SRI and LRI protocols are listed in Table 2. Large-sized decrease in the reverse-Stroop interference score was observed immediately after SRI protocol (*d*=0.94 and 0.82, respectively, vs. baseline and pre-exercise), whereas moderate-sized decrease in the reverse-Stroop interference score was observed immediately after LRI protocol (*d*=0.62 and 0.66, respectively, vs. baseline and pre-exercise). Moreover, significant decreases in the reverse-Stroop interference score were observed from 10 to 30min after SRI protocol (all *p*s<0.05, *d*=1.11–1.42 vs. baseline and/or pre-exercise); by contrast, no such significant decrease was observed after LRI protocol (all *p*s>0.05, *d*=−0.01 to 0.62). Furthermore, the reverse-Stroop interference scores 20 and 30min after SRI protocol were significantly lower than those after LRI protocol (both *p*s<0.01, *d*=1.05 and 0.90, respectively).

**Table 2:** Effect size and 95% CI for comparison of the reverse-Stroop interference score between before and after SRI and LRI protocols.

	SRI		LRI	
	Baseline	Pre-EX	Baseline	Pre-EX
Post-EX 0min	0.94 [0.29 to 1.59]	0.82 [0.17 to 1.46]^*p* =0.074^	0.62 [−0.01 to 1.26]	0.66 [0.03 to 1.30]
Post-EX 10min	1.42 [0.73 to 2.12]^*^	1.18 [0.51 to 1.85]^*^	0.59 [−0.04 to 1.23]	0.62 [−0.01 to 1.26]
Post-EX 20min	1.26 [0.58 to 1.93]^*^	1.02 [0.36 to 1.67]	0.19 [−0.43 to 0.81]	0.18 [−0.44 to 0.80]
Post-EX 30min	1.11 [0.44 to 1.77]^*^	0.92 [0.27 to 1.57]	0.02 [−0.60 to 0.64]	−0.01 [−0.63 to 0.61]

Values are presented as Cohen’s d [95% CI]. Time points at baseline and Pre-EX were designated “before exercise.” Time points at immediately after exercise (i.e., Post-EX 0min) and during the 30-min post-exercise recovery period (i.e., Post-EX 10, 20, and 30min) were designated “after exercise.”

* Significant difference (all *p*s<0.05) between before and after exercise.

### Changes in Psychological Conditions for the CWST Throughout Experimental Session

Changes in psychological conditions for the CWST throughout SRI and LRI sessions are shown in Table 3. Arousal analysis revealed a significant main effect for time (*F*_(2.29, 43.51)_=15.77, *p*<0.001, *η*_p_^2^=0.45); however, there were no significant main effect for condition (*F*_(1, 19)_=0.01, *p*=0.939, *η_p_*^2^<0.01) and no significant interaction effect (*F*_(3.01, 58.27)_=1.31, *p*=0.280, *η_p_*^2^=0.07). *Post hoc* comparison for this time main effect indicated that arousal significantly increased immediately and 10min after exercise compared with that before exercise (all *p*s<0.05 vs. baseline and/or pre-exercise). Analyses of mental fatigue and motivation revealed significant effects for time (mental fatigue: *F*_(2.37, 45.07)_=16.98, *p*<0.001, *η*_p_^2^=0.47; motivation: *F*_(2.48, 47.10)_=4.94, *p*=0.007, *η*_p_^2^=0.21); however, there were no significant main effects for condition (mental fatigue: *F*_(1, 19)_=3.67, *p*=0.071, *η*_p_^2^=0.16; motivation: *F*_(1, 19)_=0.83, *p*=0.373, *η*_p_^2^=0.04) and no significant interaction effects (mental fatigue: *F*_(5, 95)_=0.89, *p*=0.490, *η*_p_^2^=0.05; motivation: *F*_(2.34, 44.54)_=0.69, *p*=0.531, *η*_p_^2^=0.04). *Post hoc* comparison of the time main effect for mental fatigue indicated that it significantly increased immediately after exercise compared with that before exercise (both *p*s<0.001 vs. baseline and pre-exercise). The increased mental fatigue remained significant until 20min after exercise (all *p*s<0.01 vs. baseline and pre-exercise). *Post hoc* comparison of the time main effect for motivation indicated it did not significantly differ among all six time points. Concentration analysis revealed no significant main effects for condition (*F*_(1, 19)_=0.34, *p*=0.569, *η*_p_^2^=0.02) and time (*F*_(2.59, 49.24)_=1.24, *p*=0.305, *η*_p_^2^=0.06) and no significant interaction effect (*F*_(5, 95)_=1.30, *p*=0.271, *η*_p_^2^=0.06).

**Table 3:** Psychological conditions for the CWST throughout SRI and LRI sessions.

	Time points						*p*		
	Baseline	Pre-EX	Post-EX 0	Post-EX 10	Post-EX 20	Post-EX 30	Time	Condition	Interaction
**Arousal (scale)**									
SRI	2.5 ± 0.2	2.7 ± 0.1	3.6 ± 0.2^a^^,^^b^	3.1 ± 0.2	2.9 ± 0.2^c^	2.7 ± 0.2^c^	0.939	**<0.001**	0.280
LRI	2.3 ± 0.2	2.6 ± 0.2	3.6 ± 0.2^a^^,^^b^	3.1 ± 0.2	3.0 ± 0.2^c^	3.0 ± 0.2			
**Mental fatigue (mm)**									
SRI	22.7 ± 5.0	27.3 ± 5.1	49.9 ± 5.1^a^^,^^b^	40.7 ± 5.8^a^	37.2 ± 4.8	32.1 ± 4.6	0.071	**<0.001**	0.490
LRI	16.2 ± 3.4	20.7 ± 4.1	38.6 ± 4.3^a^^,^^b^	32.1 ± 4.3^a^^,^^b^	33.3 ± 5.1^a^^,^^b^	27.4 ± 4.8			
**Motivation (mm)**									
SRI	72.0 ± 3.8	69.1 ± 5.0	79.1 ± 3.0	73.1 ± 4.7	66.6 ± 5.0	66.7 ± 5.3	0.373	0.007	0.531
LRI	68.0 ± 4.8	65.7 ± 4.4	75.7 ± 3.5	69.3 ± 4.3	68.0 ± 4.5	65.9 ± 5.0			
**Concentration (mm)**									
SRI	68.6 ± 5.3	65.4 ± 5.4	66.1 ± 5.0	67.7 ± 5.7	61.7 ± 4.0	61.7 ± 5.6	0.569	0.305	0.271
LRI	61.0 ± 4.9	60.4 ± 4.5	69.2 ± 4.6	62.5 ± 4.5	60.5 ± 4.2	60.0 ± 4.2			

Values are presented as mean±standard error of the mean. Bold values of *p* indicate significant main time effects for arousal and mental fatigue.

a *p*<0.05 vs. Baseline.

b *p*<0.05 vs. Pre-EX.

c *p*<0.05 vs. Post-EX 0.

## Discussion

The results of this study showed that large-sized decreases in the reverse-Stroop interference score, which represent improved IC, were observed throughout 30min after SRI protocol. In contrast, moderate-sized decreases in the reverse-Stroop interference score were observed immediately and 10min after LRI protocol. Moreover, significant decreases in the reverse-Stroop interference score were observed from 10 to 30min after SRI protocol, whereas no such significant difference for the reverse-Stroop interference score was observed after LRI protocol. Furthermore, the degree of decreases in the reverse-Stroop interference score throughout the 30-min post-exercise recovery period was greater following SRI protocol than following LRI protocol, as significant interaction effect was observed. Therefore, these present findings suggest that the SRI protocol can effectively improve IC following low-intensity resistance exercise compared to the LRI protocol.

In this study, the reverse-Stroop interference scores immediately after SRI and LRI protocols were decreased by ≥38.8 and 23.2%, respectively, compared to those before each exercise (i.e., baseline and pre-exercise). Furthermore, the difference in the reverse-Stroop interference scores between before and immediately after exercise showed large effect size (i.e., *d*=0.94 and 0.82, respectively, vs. baseline and pre-exercise) for SRI protocol, whereas it showed moderate effect size for LRI protocol (i.e., *d*=0.62 and 0.66, respectively, vs. baseline and pre-exercise). These present findings suggest that the immediate effect on IC improvements following low-intensity resistance exercise is greater with SRI protocol than with LRI protocol. Our previous study obtained that the immediate improvement in post-exercise IC was greater for high-intensity resistance exercise than for low-intensity resistance exercise (Tsukamoto et al., 2017a), suggesting that exercise intensity may play an important role in determining the degree of the immediate effect on the post-exercise IC improvement. Nevertheless, because this study used a same number of repetitions per set, work volume (i.e., intensity×total repetitions) is higher for high-intensity resistance exercise than for low-intensity resistance exercise. To address this issue, in a recent study, we compared the effect of high-volume low-intensity resistance exercise (i.e., 35% 1-RM, 20 repetitions/set, six sets) with a same work volume to that of high-intensity resistance exercise (i.e., 70% 1-RM, 10 repetitions/set, six sets) on post-exercise IC (Tomoo et al., 2020). We found that the IC improvement immediately after high-volume low-intensity resistance exercise was comparable to that immediately after high-intensity resistance exercise (≥34.6 and 38.8%, respectively, for the decreasing rate of the reverse-Stroop interference score; Tomoo et al., 2020). Considering these previous findings, it can be inferred that work load, rather than intensity, may be a more important variable in determining the degree of the immediate effect on the post-exercise IC improvement. Therefore, the present findings suggest that the inter-set rest interval length may also be an important variable for IC improvement immediately after resistance exercise, particularly low-intensity resistance exercise.

Our previous study found that although the magnitude of improvement in IC immediately after aerobic exercise (i.e., immediate effect) did not differ between high-intensity interval exercise and moderate-intensity continuous exercise, the duration of significant improvements in IC throughout the 30-min post-exercise recovery period (i.e., sustainable effect) was longer after high-intensity interval exercise than after moderate-intensity continuous exercise (Tsukamoto et al., 2016a). Considering this finding, in addition to the immediate effect, the sustainable effect may be important to understand the difference in the potential effect on post-exercise IC improvements among exercise protocols (Tsukamoto et al., 2016a, 2017b, 2018; Tanaka et al., 2018; Sugimoto et al., 2020; Tomoo et al., 2020). We and others have previously reported the sustainable effects of resistance exercise on post-exercise IC improvements (Brush et al., 2016; Johnson et al., 2016; Tomoo et al., 2020). Of these studies, Johnson et al. (2016) reported that although IC improved immediately after both moderate-intensity whole-body resistance exercises (i.e., 60% 1-RM) with 10- and 30-min durations, the improved IC reversed at the 30-min post-exercise recovery period. Their findings suggest that duration of IC improvements following resistance exercise may be less than 30min. Nevertheless, the study by Johnson et al. (2016) recruited older individuals, and therefore, the sustainable effect on post-exercise IC improvements may be limited in this population. Our previous studies identified that the sustainable effect on post-exercise IC improvements is observed until 20min after localized knee extensor high-intensity resistance exercise (Tomoo et al., 2020). A similar trend was also observed for the knee extensor high-volume low-intensity resistance exercise (Tomoo et al., 2020). Furthermore, in the present study, we obtained that significant decreases in the reverse-Stroop interference score induced by the knee extensor low-intensity resistance exercise were observed from 10 to 30min following SRI protocol, but not following LRI protocol. Therefore, the present findings suggest that in addition to the high-intensity resistance exercise and high-volume low-intensity resistance exercise, low-intensity resistance exercise with SRI may have a certain sustainable effect on post-exercise IC improvements. Additionally, the SRI protocol can effectively enhance both immediate and sustainable effects on low-intensity resistance exercise-induced IC improvements compared to the LRI protocol.

During exercise, lactate acts as an important energy substrate, instead of glucose, in the human brain (van Hall et al., 2009). Our previous studies demonstrated that aerobic exercise-induced IC improvements are related to circulating lactate levels (Tsukamoto et al., 2016b; Hashimoto et al., 2018), potentially by increasing cerebral lactate metabolism (Hashimoto et al., 2018). In the present study, we obtained that low-intensity resistance exercise-induced increase in blood lactate levels was greater following SRI protocol than following LRI protocol, as significant interaction effect was observed. This present finding corroborates the results of previous study (Kraemer et al., 1990, 1993). Therefore, greater circulating lactate secretion for SRI protocol than LRI protocol may contribute to our understanding of the difference in the degrees of post-exercise IC improvements between the two protocols.

In this study, the EMG activities of three quadriceps femoris muscles (i.e., the vastus lateralis, vastus medialis, and rectus femoris) were increased in the final set compared to the first set during SRI protocol, but not during LRI protocol. Furthermore, the quadriceps vastus lateralis EMG activity during the final set was higher with SRI protocol than with LRI protocol. These results of the quadriceps femoris EMG activities may be associated with greater IC improvements following SRI protocol than LRI protocol, which is consistent with the results of our previous study that determined the difference in the immediate effect of post-exercise IC improvement between high- and low-intensity resistance exercises when repetition per set was the same between the protocols. Previous studies have reported that the increase in the EMG activity of exercising muscle(s) due to increased force output during resistance exercise was in proportional to the increase in cerebral neural activity (Dai et al., 2001; van Duinen et al., 2008), which may be based on the interaction between peripheral and central nervous systems. The cerebral neural activation is probably the most prominent determinant of cognitive function, including IC (Richeson et al., 2003; Hyodo et al., 2016). Therefore, greater neural activations of the exercising muscles during SRI protocol than LRI protocol may also contribute to our understanding of the difference in the degrees of post-exercise IC improvements between the two protocols.

The magnitudes of psychological and perceptual responses during exercise may be associated with increasing the cerebral neural activity and improving the cognitive function (Berchicci et al., 2013; Byun et al., 2014; Fontes et al., 2015). Byun et al. (2014) reported that aerobic exercise-induced increase in arousal is correlated with increased cerebral neural activity and improved cognitive function. Unexpectedly, in the present study, we obtained that change in arousal throughout experimental session did not differ between SRI and LRI protocols. In contrast, increases in perceived exertion parameters (i.e., RPE and leg discomfort) during low-intensity resistance exercise were greater with SRI protocol than with LRI protocol. Previous studies have reported that increase in RPE during aerobic exercise may be associated with increased cerebral neural activity and improved cognitive function (Berchicci et al., 2013; Fontes et al., 2015). Therefore, greater perceptual response, such as perceived exertion, during SRI protocol than LRI protocol may contribute, at least partially, to our understanding of the difference in the degrees of post-exercise IC improvements between the two protocols.

This study employed 1-min inter-set rest intervals for SRI protocol, as in many previous studies (see a review by de Salles et al., 2009). Nevertheless, some previous studies used inter-set rest intervals shorter than 1min (i.e., 30s; Takarada and ishii, 2002; Goto et al., 2004). Furthermore, we employed 40% 1-RM for carrying out low-intensity resistance exercise based on our previous study (Tsukamoto et al., 2017a), while most previous studies examining resistance exercise with SRI used exercise loads heavier than 40% 1-RM (see a review by de Salles et al., 2009). Further studies are needed to determine the most effective method of SRI protocol on post-exercise IC improvements.

Previous studies have reported that increases in circulating endocrine responses induced by acute bout of resistance exercise were greater with SRI protocol than with LRI protocol (Kraemer et al., 1990, 1993; Goto et al., 2004). A series of studies by Kraemer et al. (1990, 1993) reported that resistance exercise-induced increases in growth hormone were greater following SRI protocol than following LRI protocol. The increased growth hormone secretion induced by acute resistance exercise may contribute to improve cognitive function and brain health (Sonntag et al., 2005), potentially *via* enhancing basal secretions of insulin-like growth factor 1, which possibly mediate the expression of brain-derived neurotrophic factor in the brain (Mattson et al., 2004). Although the long-term training effects of resistance exercise with SRI on cognitive function and brain health are unknown, these previous findings suggest that the SRI protocol has greater potential to improve these parameters compared to the LRI protocol. Furthermore, in a series of studies by Kraemer et al. (1990, 1993), they found that SRI protocols with increased repetitions per set (i.e., total repetitions) were more effective in enhancing circulating endocrine responses (e.g., growth hormone) than other SRI protocols, including those with increased exercise intensity. The increases in the loads on the body, particularly on the cardiovascular and musculoskeletal systems, during resistance exercise may be lesser with an increase in the total number of repetitions than with an increase in the exercise intensity when the work volume was matched (Tomoo et al., 2020). Therefore, increasing the total number of repetitions compared to increasing the exercise intensity appears to be more useful for the creation of safe and effective resistance programs for older individuals and patients with chronic diseases. To elucidate our proposal, further studies are needed to determine the long-term training effect of the most effective SRI protocol on cognitive function and brain health.

Byun et al. (2014) reported that a 10-min duration of low-intensity aerobic exercise could improve immediate post-exercise IC. Nevertheless, we have previously reported that the degree of sustainable effect on post-exercise IC improvements induced by aerobic exercise may depend on the duration of the exercise (Tsukamoto et al., 2017b). Thus, the sustainable effect on IC improvements following the 10-min low-intensity aerobic exercise may not be suitable. In contrast to this aerobic exercise protocol, the low-intensity resistance exercise protocol with SRI employed in the present study was completed in approximately 7min. Despite this shorter exercise time, we found that both immediate and sustainable effects on post-exercise IC improvements induced by the SRI protocol may be greater than those induced by traditional aerobic exercise protocols (e.g., low- or moderate-intensity aerobic continuous exercises for 20- and/or 40-min durations) and other resistance exercise protocols (e.g., low-intensity resistance exercise with LRI for 17min), which have exercise durations/times longer than those of the SRI protocol. Therefore, the SRI protocol may be useful for creating resistance exercise programs to effectively improve cognitive function with a shorter exercise time.

A review by Pontifex et al. (2019) has suggested that the control condition such as sitting at rest should be included the main experiment in order to increase the rigor of investigating the acute effect of exercise on cognitive function. In our previous studies, we have reported no effect of sitting rest on IC (Tsukamoto et al., 2017a,b; Sugimoto et al., 2020), which may indicate that the IC improvements following exercise is not attributable to learning effect. Furthermore, in a preliminary procedure of this study, we also demonstrated that a 17-min sitting rest did not change the IC throughout the same period of the main experiment. However, we did not include the control condition in the experimental protocols of the main study, which is a major limitation of this study. Therefore, the main result of this study is limited to determining the difference in the degree of post-exercise IC improvements between SRI and LRI protocols.

Another major limitation of in this study is that we recruited only healthy young males; therefore, whether the present findings can be generalized to other populations is unclear. In particular, we did not recruit young females, because of their menstrual cycles, as this may affect muscular performances, especially force output and fatigue (Sarwar et al., 1996), during resistance exercise (McNulty et al., 2020). Furthermore, the menstrual cycle of young females may affect their basal (i.e., resting) CWST performances (Hatta and Nagaya, 2009), potentially by modulating circulating cognition-related hormones/cytokines secretions (e.g., brain-derived neurotrophic factor) and cerebral blood flow and neural activity (Brackley et al., 1999; Pluchino et al., 2009; Otomo et al., 2020), which may be important factors in mediating cognitive performance (Richeson et al., 2003; Lucas et al., 2012; Whiteman et al., 2014; Hyodo et al., 2016). Considering these physiological factors, we have not recruited young females in a series of our studies (e.g., Tomoo et al., 2020). Nevertheless, in a recent study, Dirk et al. (2020) reported that the improvement in the antisaccade task-measured executive function immediately after a 20-min moderate-intensity aerobic exercise did not differ between the follicular and luteal menstrual cycle phases in young females. Their findings suggest that the menstrual cycle may not affect the degree of improvements in post-exercise executive function, including IC, in this population. Additionally, effective resistance exercise programs to improve cognitive function are generally more important for older individuals and patients with chronic diseases than for healthy young individuals. To utilize the present findings in clinical settings, further studies are needed to determine the effect of SRI protocol on post-exercise cognitive function improvements in various populations (e.g., young females, older individuals, and patients with chronic diseases).

## Conclusion

This study demonstrated that the SRI protocol was more useful in improving post-exercise IC, potentially *via* greater circulating lactate secretion, neural activity of the exercising muscle, and perceptual response, compared to the LRI protocol. Therefore, we suggest that the inter-set rest interval length may be an important variable for determining the degree of cognitive function improvements following low-intensity resistance exercise in healthy young males.

## Data Availability Statement

The raw data supporting the conclusions of this article will be made available by the authors, without undue reservation.

## Ethics Statement

The studies involving human participants were reviewed and approved by the Ethics Committee of Ritsumeikan University. The patients/participants provided their written informed consent to participate in this study.

## Author Contributions

KT and TadaS conceived and designed the experiment, analyzed data, and wrote the manuscript. KT, TadaS, KD, TakeS, and EM performed the experiments. KT, TadaS, KD, TakeS, EM, HT, ST, TH, and TI interpreted the results of the experiments. TadaS, HT, ST, TH, and TI edited and revised the manuscript. All authors contributed to the article and approved the submitted version.

## Funding

This study was supported in part by Center of Innovation Program from Japan Science and Technology Agency (#JPMJCE1306 to TadaS and TI; #JPMJCE1301 to ST).

## Conflict of Interest

The authors declare that the research was conducted in the absence of any commercial or financial relationships that could be construed as a potential conflict of interest.

## Publisher’s Note

All claims expressed in this article are solely those of the authors and do not necessarily represent those of their affiliated organizations, or those of the publisher, the editors and the reviewers. Any product that may be evaluated in this article, or claim that may be made by its manufacturer, is not guaranteed or endorsed by the publisher.

## References

[ref1] Ai J. Y. Chen F. T. Hsieh S. S. Kao S. C. Chen A. G. Hung T. M.. (2021). The effect of acute high-intensity interval training on executive function: a systematic review. Int. J. Environ. Res. Public Health 18:3593. doi: 10.3390/ijerph18073593, PMID: 33808399 PMC8037758

[ref2] American College of Sports Medicine (2009). American college of sports medicine position stand. Progression models in resistance training for healthy adults. Med. Sci. Sports Exerc. 41, 687–708. doi: 10.1249/MSS.0b013e3181915670, PMID: 19204579

[ref3] Atkinson H. H. Cesari M. Kritchevsky S. B. Penninx B. W. Fried L. P. Guralnik J. M.. (2005). Predictors of combined cognitive and physical decline. J. Am. Geriatr. Soc. 53, 1197–1202. doi: 10.1111/j.1532-5415.2005.53362.x 16108938

[ref4] Berchicci M. Menotti F. Macaluso A. Di Russo F. (2013). The neurophysiology of central and peripheral fatigue during sub-maximal lower limb isometric contractions. Front. Hum. Neurosci. 7:135. doi: 10.3389/fnhum.2013.00135, PMID: 23596408 PMC3625743

[ref5] Borg G. A. (1982). Psychophysical bases of perceived exertion. Med. Sci. Sports Exerc. 14, 377–381. PMID: 7154893

[ref6] Brackley K. J. Ramsay M. M. Broughton Pipkin F. Rubin P. C. (1999). The effect of the menstrual cycle on human cerebral blood flow: studies using Doppler ultrasound. Ultrasound Obstet. Gynecol. 14, 52–57. doi: 10.1046/j.1469-0705.1999.14010052.x, PMID: 10461339

[ref7] Brush C. J. Olson R. L. Ehmann P. J. Osovsky S. Alderman B. L. (2016). Dose–response and time course effects of acute resistance exercise on executive function. J. Sport Exerc. Psychol. 38, 396–408. doi: 10.1123/jsep.2016-0027, PMID: 27385719

[ref8] Byun K. Hyodo K. Suwabe K. Ochi G. Sakairi Y. Kato M.. (2014). Positive effect of acute mild exercise on executive function via arousal-related prefrontal activations: an fNIRS study. NeuroImage 98, 336–345. doi: 10.1016/j.neuroimage.2014.04.067, PMID: 24799137

[ref9] Chang K. V. Hsu T. H. Wu W. T. Huang K. C. Han D. S. (2016). Association between sarcopenia and cognitive impairment: a systematic review and meta-analysis. J. Am. Med. Dir. Assoc. 17, 1164.e7–1164.e15. doi: 10.1016/j.jamda.2016.09.013 27816484

[ref10] Chang Y. K. Labban J. D. Gapin J. I. Etnier J. L. (2012). The effects of acute exercise on cognitive performance: a meta-analysis. Brain Res. 1453, 87–101. doi: 10.1016/j.brainres.2012.02.068, PMID: 22480735

[ref11] Cohen J. A. (1992). A power primer. Psychol. Bull. 112, 155–159. doi: 10.1037/0033-2909.112.1.155, PMID: 19565683

[ref12] Coxon J. P. Stinear C. M. Byblow W. D. (2007). Selective inhibition of movement. J. Neurophysiol. 97, 2480–2499. doi: 10.1152/jn.01284.2006, PMID: 17251361

[ref13] Dai T. H. Liu J. Z. Sahgal V. Brown R. W. Yue G. H. (2001). Relationship between muscle output and functional MRI-measured brain activation. Exp. Brain Res. 140, 290–300. doi: 10.1007/s002210100815, PMID: 11681304

[ref15] DeCarli C. (2003). Mild cognitive impairment: prevalence, prognosis, aetiology, and treatment. Lancet Neurol. 2, 15–21. doi: 10.1016/s1474-4422(03)00262-x 12849297

[ref14] de Salles B. F. Simão R. Miranda F. Novaes Jda S. Lemos A. Willardson J. M. (2009). Rest interval between sets in strength training. Sports Med. 39, 765–777. doi: 10.2165/11315230-000000000-00000, PMID: 19691365

[ref16] Dirk K. L. Belfry G. R. Heath M. (2020). Exercise and executive function during follicular and luteal menstrual cycle phases. Med. Sci. Sports Exerc. 52, 919–927. doi: 10.1249/MSS.0000000000002192, PMID: 31652244

[ref17] Faul F. Erdfelder E. Lang A. G. Buchner A. (2007). G*power 3: a flexible statistical power analysis program for the social, behavioral, and biomedical sciences. Behav. Res. Methods 39, 175–191. doi: 10.3758/BF03193146, PMID: 17695343

[ref18] Fontes E. B. Okano A. H. De Guio F. Schabort E. J. Min L. L. Basset F. A.. (2015). Brain activity and perceived exertion during cycling exercise: an fMRI study. Br. J. Sports Med. 49, 556–560. doi: 10.1136/bjsports-2012-091924, PMID: 23729175

[ref19] Goto K. Nagasawa M. Yanagisawa O. Kizuka T. Ishii N. Takamatsu K. (2004). Muscular adaptations to combinations of high- and low-intensity resistance exercises. J. Strength Cond. Res. 18, 730–737. doi: 10.1519/R-13603.1, PMID: 15574075

[ref20] Hashimoto T. Tsukamoto H. Takenaka S. Olesen N. D. Petersen L. G. Sørensen H.. (2018). Maintained exercise-enhanced brain executive function related to cerebral lactate metabolism in men. FASEB J. 32, 1417–1427. doi: 10.1096/fj.201700381RR, PMID: 29127193

[ref21] Hatta T. Nagaya K. (2009). Menstrual cycle phase effects on memory and Stroop task performance. Arch. Sex. Behav. 38, 821–827. doi: 10.1007/s10508-008-9445-7, PMID: 19130208

[ref22] Hyodo K. Dan I. Kyutoku Y. Suwabe K. Byun K. Ochi G.. (2016). The association between aerobic fitness and cognitive function in older men mediated by frontal lateralization. NeuroImage 125, 291–300. doi: 10.1016/j.neuroimage.2015.09.062, PMID: 26439424

[ref23] Ikeda Y. Hirata S. Okuzumi H. Kokubun M. (2010). Features of Stroop and reverse-Stroop interference: analysis by response modality and evaluation. Percept. Mot. Skills 110, 654–660. doi: 10.2466/pms.110.2.654-660, PMID: 20499573

[ref24] Johnson L. Addamo P. K. Selva Raj I. Borkoles E. Wyckelsma V. Cyarto E.. (2016). An acute bout of exercise improves the cognitive performance of older adults. J. Aging Phys. Act. 24, 591–598. doi: 10.1123/japa.2015-0097, PMID: 26964644

[ref25] Kraemer W. J. Fleck S. J. Dziados J. E. Harman E. A. Marchitelli L. J. Gordon S. E.. (1993). Changes in hormonal concentrations after different heavy-resistance exercise protocols in women. J. Appl. Physiol. 75, 594–604. doi: 10.1152/jappl.1993.75.2.594, PMID: 8226457

[ref26] Kraemer W. J. Marchitelli L. Gordon S. E. Harman E. Dziados J. E. Mello R.. (1990). Hormonal and growth factor responses to heavy resistance exercise protocols. J. Appl. Physiol. 69, 1442–1450. doi: 10.1152/jappl.1990.69.4.1442, PMID: 2262468

[ref27] Lucas S. J. Ainslie P. N. Murrell C. J. Thomas K. N. Franz E. A. Cotter J. D. (2012). Effect of age on exercise-induced alterations in cognitive executive function: relationship to cerebral perfusion. Exp. Gerontol. 47, 541–551. doi: 10.1016/j.exger.2011.12.002, PMID: 22230488

[ref28] Ludyga S. Gerber M. Pühse U. Looser V. N. Kamijo K. (2020). Systematic review and meta-analysis investigating moderators of long-term effects of exercise on cognition in healthy individuals. Nat. Hum. Behav. 4, 603–612. doi: 10.1038/s41562-020-0851-8, PMID: 32231280

[ref29] Mattson M. P. Maudsley S. Martin B. (2004). A neural signaling triumvirate that influences ageing and age-related disease: insulin/IGF-1, BDNF and serotonin. Ageing Res. Rev. 3, 445–464. doi: 10.1016/j.arr.2004.08.001, PMID: 15541711

[ref30] McMorris T. Hale B. J. (2012). Differential effects of differing intensities of acute exercise on speed and accuracy of cognition: a meta-analytical investigation. Brain Cogn. 80, 338–351. doi: 10.1016/j.bandc.2012.09.001, PMID: 23064033

[ref31] McNulty K. L. Elliott-Sale K. J. Dolan E. Swinton P. A. Ansdell P. Goodall S.. (2020). The effects of menstrual cycle phase on exercise performance in eumenorrheic women: a systematic review and meta-analysis. Sports Med. 50, 1813–1827. doi: 10.1007/s40279-020-01319-3, PMID: 32661839 PMC7497427

[ref32] Moreau D. Chou E. (2019). The acute effect of high-intensity exercise on executive function: a meta-analysis. Perspect. Psychol. Sci. 14, 734–764. doi: 10.1177/1745691619850568, PMID: 31365839

[ref33] Northey J. M. Cherbuin N. Pumpa K. L. Smee D. J. Rattray B. (2018). Exercise interventions for cognitive function in adults older than 50: a systematic review with meta-analysis. Br. J. Sports Med. 52, 154–160. doi: 10.1136/bjsports-2016-096587, PMID: 28438770

[ref34] Oberste M. Javelle F. Sharma S. Joisten N. Walzik D. Bloch W.. (2019). Effects and moderators of acute aerobic exercise on subsequent interference control: a systematic review and meta-analysis. Front. Psychol. 10:2616. doi: 10.3389/fpsyg.2019.02616, PMID: 31824387 PMC6881262

[ref35] Otomo M. Harada M. Abe T. Matsumoto Y. Abe Y. Kanazawa Y.. (2020). Reproducibility and variability of quantitative cerebral blood flow measured by multi-delay 3D arterial spin labeling according to sex and menstrual cycle. J. Med. Investig. 67, 321–327. doi: 10.2152/jmi.67.321, PMID: 33148909

[ref36] Ozonoff S. Strayer D. L. (1997). Inhibitory function in nonretarded children with autism. J. Autism Dev. Disord. 27, 59–77. doi: 10.1023/A:1025821222046, PMID: 9018582

[ref37] Pluchino N. Cubeddu A. Begliuomini S. Merlini S. Giannini A. Bucci F.. (2009). Daily variation of brain-derived neurotrophic factor and cortisol in women with normal menstrual cycles, undergoing oral contraception and in postmenopause. Hum. Reprod. 24, 2303–2309. doi: 10.1093/humrep/dep119, PMID: 19491202

[ref38] Pontifex M. B. McGowan A. L. Chandler M. C. Gwizdala K. L. Parks A. C. Fenn K.. (2019). A primer on investigating the after effects of acute bouts of physical activity on cognition. Psychol. Sport Exerc. 40, 1–22. doi: 10.1016/j.psychsport.2018.08.015

[ref39] Richeson J. A. Baird A. A. Gordon H. L. Heatherton T. F. Wyland C. L. Trawalter S.. (2003). An fMRI investigation of the impact of interracial contact on executive function. Nat. Neurosci. 6, 1323–1328. doi: 10.1038/nn1156, PMID: 14625557

[ref40] Ruiz J. R. Sui X. Lobelo F. Morrow J. R. Jr. Jackson A. W. Sjöström M.. (2008). Association between muscular strength and mortality in men: prospective cohort study. BMJ 337:a439. doi: 10.1136/bmj.a439, PMID: 18595904 PMC2453303

[ref41] Sarwar R. Niclos B. B. Rutherford O. M. (1996). Changes in muscle strength, relaxation rate and fatiguability during the human menstrual cycle. J. Physiol. 493, 267–272. doi: 10.1113/jphysiol.1996.sp021381, PMID: 8735711 PMC1158967

[ref42] Smith E. E. Jonides J. (1999). Storage and executive processes in the frontal lobes. Science 283, 1657–1661. doi: 10.1126/science.283.5408.1657, PMID: 10073923

[ref43] Sonntag W. E. Ramsey M. Carter C. S. (2005). Growth hormone and insulin-like growth factor-1 (IGF-1) and their influence on cognitive aging. Ageing Res. Rev. 4, 195–212. doi: 10.1016/j.arr.2005.02.001, PMID: 16024298

[ref44] Suga T. Okita K. Morita N. Yokota T. Hirabayashi K. Horiuchi M.. (2009). Intramuscular metabolism during low-intensity resistance exercise with blood flow restriction. J. Appl. Physiol. 106, 1119–1124. doi: 10.1152/japplphysiol.90368.2008, PMID: 19213931

[ref45] Sugimoto T. Suga T. Tomoo K. Dora K. Mok E. Tsukamoto H.. (2021). Blood flow restriction improves executive function after walking. Med. Sci. Sports Exerc. 53, 131–138. doi: 10.1249/MSS.0000000000002446, PMID: 32694372

[ref46] Sugimoto T. Suga T. Tsukamoto H. Tomoo K. Dora K. Hashimoto T.. (2020). Effect of repeated bouts versus a single bout of moderate-intensity exercise on postexercise inhibitory control. Phys. Rep. 8:e14528. doi: 10.14814/phy2.14528, PMID: 32776468 PMC7415913

[ref47] Svebak S. Murgatroyd S. (1985). Metamotivational dominance: a multimethod validation of reversal theory constructs. J. Pers. Soc. Psychol. 48, 107–116. doi: 10.1037/0022-3514.48.1.107

[ref48] Takarada Y. Ishii N. (2002). Effects of low-intensity resistance exercise with short interset rest period on muscular function in middle-aged women. J. Strength Cond. Res. 16, 123–128. PMID: 11834117

[ref49] Tanaka D. Tsukamoto H. Suga T. Takenaka S. Hamaoka T. Hashimoto T.. (2018). Self-selected music-induced reduction of perceived exertion during moderate-intensity exercise does not interfere with postexercise improvements in inhibitory control. Physiol. Behav. 194, 170–176. doi: 10.1016/j.physbeh.2018.05.030, PMID: 29807054

[ref50] Tomoo K. Suga T. Sugimoto T. Tanaka D. Shimoho K. Dora K.. (2020). Work volume is an important variable in determining the degree of inhibitory control improvements following resistance exercise. Phys. Rep. 8:e14527. doi: 10.14814/phy2.14527, PMID: 32776493 PMC7415911

[ref51] Tomporowski P. D. (2003). Effects of acute bouts of exercise on cognition. Acta Psychol. 112, 297–324. doi: 10.1016/S0001-6918(02)00134-8, PMID: 12595152

[ref53] Tsukamoto H. Suga T. Ishibashi A. Takenaka S. Tanaka D. Hirano Y.. (2018). Flavanol-rich cocoa consumption enhances exercise-induced executive function improvements in humans. Nutrition 46, 90–96. doi: 10.1016/j.nut.2017.08.017, PMID: 29290363

[ref54] Tsukamoto H. Suga T. Takenaka S. Takeuchi T. Tanaka D. Hamaoka T.. (2017a). An acute bout of localized resistance exercise can rapidly improve inhibitory control. PLoS One 12:e0184075. doi: 10.1371/journal.pone.0184075 28877232 PMC5587287

[ref55] Tsukamoto H. Suga T. Takenaka S. Tanaka D. Takeuchi T. Hamaoka T.. (2016a). Greater impact of acute high-intensity interval exercise on postexercise executive function compared to moderate-intensity continuous exercise. Physiol. Behav. 155, 224–230. doi: 10.1016/j.physbeh.2015.12.021 26723268

[ref56] Tsukamoto H. Suga T. Takenaka S. Tanaka D. Takeuchi T. Hamaoka T.. (2016b). Repeated high-intensity interval exercise shortens the positive effect on executive function during postexercise recovery in healthy young males. Physiol. Behav. 160, 26–34. doi: 10.1016/j.physbeh.2016.03.029 27060507

[ref57] Tsukamoto H. Takenaka S. Suga T. Tanaka D. Takeuchi T. Hamaoka T.. (2017b). Impact of exercise intensity and duration on postexercise executive function. Med. Sci. Sports Exerc. 49, 774–784. doi: 10.1249/MSS.0000000000001155 27846044

[ref58] van Duinen H. Renken R. Maurits N. M. Zijdewind I. (2008). Relation between muscle and brain activity during isometric contractions of the first dorsal interosseus muscle. Hum. Brain Mapp. 29, 281–299. doi: 10.1002/hbm.20388, PMID: 17394210 PMC6870705

[ref59] van Hall G. Strømstad M. Rasmussen P. Jans O. Zaar M. Gam C.. (2009). Blood lactate is an important energy source for the human brain. J. Cereb. Blood Flow Metab. 29, 1121–1129. doi: 10.1038/jcbfm.2009.35, PMID: 19337275

[ref60] Whiteman A. S. Young D. E. He X. Chen T. C. Wagenaar R. C. Stern C. E.. (2014). Interaction between serum BDNF and aerobic fitness predicts recognition memory in healthy young adults. Behav. Brain Res. 259, 302–312. doi: 10.1016/j.bbr.2013.11.023, PMID: 24269495 PMC3991014

[ref61] Williams M. A. Haskell W. L. Ades P. A. Amsterdam E. A. Bittner V. Franklin B. A.. (2007). Resistance exercise in individuals with and without cardiovascular disease: 2007 update: a scientific statement from the American Heart Association Council on clinical cardiology and council on nutrition, physical activity, and metabolism. Circulation 116, 572–584. doi: 10.1161/CIRCULATIONAHA.107.185214, PMID: 17638929

